# Response to Treatment with Recombinant Human Growth Hormone (rhGH) of Short Stature Children Born Too Small for Gestational Age (SGA) in Selected Centres in Poland

**DOI:** 10.3390/jcm11113096

**Published:** 2022-05-30

**Authors:** Marta Glińska, Mieczysław Walczak, Beata Wikiera, Beata Pyrżak, Anna Majcher, Monika Paluchowska, Aneta Gawlik, Aleksandra Antosz, Marcin Kusz, Artur Bossowski, Karolina Stożek, Anna Wędrychowicz, Jerzy Starzyk, Elżbieta Petriczko

**Affiliations:** 1 Department of Pediatrics, Endocrinology, Diabetology, Metabolic Disorders and Cardiology of the Developmental Age, Pomeranian Medical University, 71-252 Szczecin, Poland; sekr.pediatrii@spsk1.szn.pl; 2 Department of Endocrinology and Diabetology of Children and Adolescents, Wroclaw Medical University, 50-368 Wroclaw, Poland; kep@usk.wroc.pl; 3 Department of Pediatrics and Endocrinology, Medical University of Warsaw, 02-091 Warsaw, Poland; endokrynologia.dsk@uckwum.pl (B.P.); amajcher@wum.edu.pl (A.M.); mpaluchowska@wum.edu.pl (M.P.); 4 Department of Paediatrics and Paediatric Endocrinology with Division of Sex Development Disorders, Medical University of Silesia, Upper Silesia Children’s Health Centre, 40-752 Katowice, Poland; endo_sk6@sum.edu.pl (A.G.); aantosz@sum.edu.pl (A.A.); marcinkusz.mk@gmail.com (M.K.); 5 Department of Pediatrics, Endocrinology, Diabetology with Cardiology Division, Medical University of Bialystok, 15-274 Bialystok, Poland; abossowski@hotmail.com (A.B.); 2klchdz@umb.edu.pl (K.S.); 6 Department of Endocrinology of Children and Young Adults, Jagiellonian University Medical College, 30-663 Krakow, Poland; endodim@cm-uj.krakow.pl (A.W.); jerzy.starzyk@uj.edu.pl (J.S.)

**Keywords:** recombinant human growth hormone, SGA, FAS, Silver-Russel syndrome

## Abstract

Short stature resulting from SGA is an obligatory indication for treatment with rhGH. The aim of the study was to assess the response to rhGH treatment in patients treated in the years 2016–2020 in six clinical centers in Poland. During the analysis, auxological data were collected, and anthropometrical parameters (Ht, SDS Ht, HV and ΔHV) were reassessed. Subgroups of patients with dysmorphic features (DYSM), fetal alcohol syndrome (FAS) and Silver-Russel syndrome (SRS) were selected. The study group consisted of 235 children (137 boys). The medium initial age was 9.08 years, and 190 patients were in the prepubertal stage. The poor response to treatment was defined as ΔHt SDS < 0.3 and/or ΔHV < 3 cm/year. Seventeen per cent of all patients after the first year and 44% after the second year met the ΔHt SDS < 0.3 criterion, and 56% during the first and 73% during the second year met the ΔHV < 3 cm/year criterion. Our data suggest that patients with SRS may show the best response to treatment, which was sustained throughout the follow-up period. The best response in all subgroups was observed during the first 12 months of therapy. Although the proportion of patients meeting the poor response criteria was high, only a few patients exceeded the 97th percentile for IGF-1 concentration during the first year of treatment. This might suggest that increasing the dose of rhGH in the second treatment year in order to sustain accelerated HV would be safe in these patients.

## 1. Introduction

The problem of short stature has for many years been one of the main issues in paediatric endocrinology. Short for gestational age (SGA) is defined as birth weight and/or length that is at least two standard deviations (SDS) below the mean for gestational age in the population [1]. Intrauterine growth retardation (IUGR) can be defined as foetuses that, for pathological reasons, have not fully exploited their growth potential [2].

It is estimated that worldwide, about 3–10% of children are born with low birth weight [3]. According to data from the Central Statistical Office, in Poland in 2019, about 21,000 neonates were born with a birth weight < 2500 g, which is 5.46% of all live births [4]. Most children with SGA accelerate growth and catch up with the population norm within the first 6–12 months of life. Approximately 10% of children do not catch up and require further diagnostic and therapeutic interventions [5]. There is growing evidence that both an unfavourable intrauterine environment and rapid postnatal weight gain in young children born with SGA contribute to the risk of developing chronic diseases and low growth in adulthood. In Poland, short stature resulting from SGA has been an obligatory indication for treatment with rhGH since 2015. According to data from the Polish National Growth Hormone Treatment Registry, 1077 children diagnosed with SGA were receiving rhGH in 2021. 

Over the years, during treatment monitoring, it has been observed that about a dozen per cent of patients (10–20%) do not meet the criteria for a good response to treatment after one year of therapy [5]. At the same time, it was noted that the response to treatment in the first year is crucial in determining the patient’s prognosis in terms of final body height and the possibility of achieving maximum benefit from the treatment provided [6].

Thus far, no clear definition of a poor (unsatisfactory) response to treatment has been established. According to the available clinical analyses, it is proposed to use various anthropometric parameters obtained during subsequent follow-up visits, e.g., growth rate expressed in cm/year (height velocity—HV), the difference in body height at baseline and body height after 12 months of treatment (ΔHt) expressed in SDS or cm [7].

In the available literature, there are few studies evaluating the response to rhGH treatment of patients with SGA in European populations, while there are no studies referring to the Polish population [8,9]. The aim of this study was to evaluate the response to treatment in short stature patients with SGA based on the population of patients from six university centres providing rhGH therapy in Poland and compare the usefulness of parameters for assessing response to treatment. 

## 2. Materials and Methods

### 2.1. Study Design

The study was observational in its nature. Auxological data of patients treated with rhGH between 2016 and 2020 at six university clinical centres (Szczecin, Katowice, Wroclaw, Krakow, Warsaw and Bialystok) were collected and analysed retrospectively. 

Patients are eligible for the Polish rhGH treatment programme dedicated to short stature children with SGA according to the unified guidelines [10]. The inclusion criteria are as follows: short stature defined by Ht < 3 percentile and ΔHV < −1 SDS according to Polish population norms; age > 4 years; GH concentration ≥ 10 ng/mL as determined by 2 of 4 growth hormone secretion stimulation tests (tests with clonidine, L-Dopa, arginine or insulin) or by a nocturnal growth hormone secretion test (at least five GH measurements); birth weight or length < −2 SD for gestational age and sex according to population norms; BA < 14 years in girls and BA < 16 years in boys (assessed by Greulich–Pyle method); exclusion of contraindications to GH therapy with contrast-enhanced CT or MRI of hypothalamic-pituitary region and exclusion of other causes of short stature. All of the criteria had to be fulfilled. 

Patients with dysmorphic features at the time of qualification for rhGH treatment undergo additional genetic testing. The diagnosis of FAS is made based on clinical findings, characteristic features of dysmorphia and a history of alcohol abuse during pregnancy. When SRS is clinically suspected, genetic testing is performed. Prior to the treatment, all of the short stature girls had to have karyotype testing provided. There are still patients with dysmorphic features who, despite extensive diagnostics, do not receive a clear diagnosis of any known genetic syndrome or chronic disease.

Once the patient is enrolled in the rhGH programme, they are obliged to attend follow-up visits—initially every 3 months during the first year of treatment, later every 6 months. During the visits, unified tests (including anthropometric measurements, laboratory testing and BA assessment) are performed. For the aim of this study, data from the qualification process as well as data from routine follow-up visits at 12 and 24 months (±3 months) were collected using a nationwide monitoring system. 

General termination criteria for the GH treatment include the following: exfoliation of the femoral head; pseudo-tumor cerebri; diabetes mellitus; diagnosis or recurrence of proliferative disease; lack of consent by the patient (or the legal guardian) to continue treatment or poor compliance; unsatisfactory treatment effect defined as ΔHV < 2 cm/year; BA > 14 years for a girl and BA > 16 years for a boy; significantly aggravated disorders of body proportions; large congenital malformations impairing basic vital functions; chromosomal aberrations associated with increased risk of proliferative diseases; elevated IGF-1 levels in relation to age and sex observed 3 months after discontinuation of growth hormone therapy.

The aim of this study was to evaluate the clinical response to rhGH treatment in the selected population. All patients included in our study group were successfully qualified for rhGH treatment according to unified guidelines. The inclusion criteria for our study were a short stature defined as Ht < 3 percentile or Ht < −2 SDS for the Polish population norms, diagnosis of SGA defined as birth weight or length < −2 SD for gestational age and sex according to population norms and treatment with growth hormone for at least 12 months. The exclusion criteria were advanced puberty during treatment and poor compliance. 

**Figure 1:**
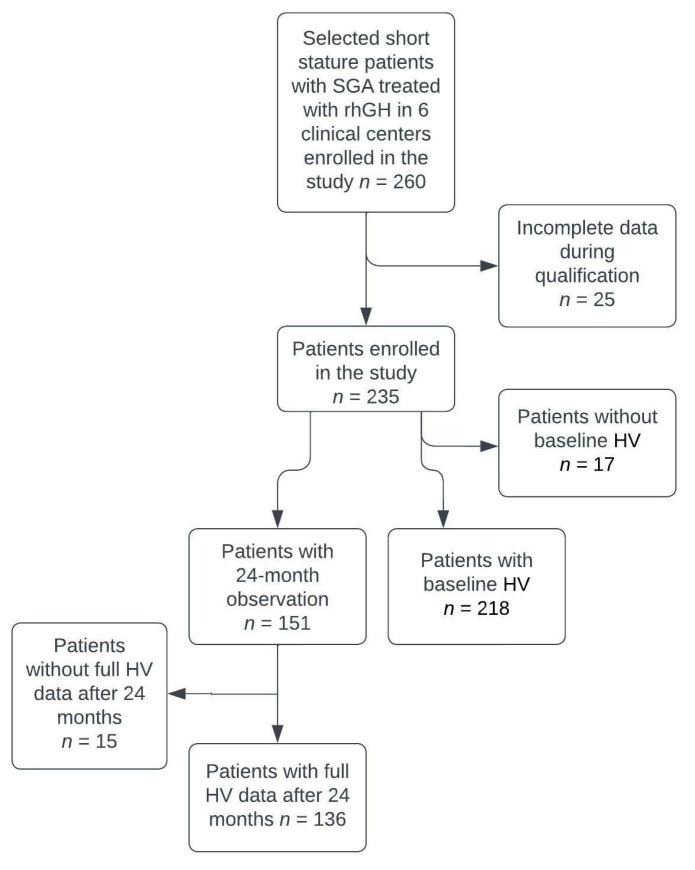
Data collection process. SGA: short for gestational age; rhGH: recombinant human growth hormone; HV: height velocity.

The data collection process is shown in Figure 1.

### 2.2. Population

The study group consisted of 235 children (137 boys). The mean age at the onset of therapy was 109 months (9.08 years). The mean bone age for the study group was 84 months (7 years). In 190 patients at the beginning of the therapy, puberty had not started yet (Tanner stage 1); 32 patients were classified as Tanner stage 2, 10 patients as stage 3 and 3 patients as stage 4. The mean birth weight expressed in SDS was −3.24. The mean body height before treatment expressed in SDS was −3.05. The mean rhGH dose at the start of treatment was 0.031 mg/kg/d. The characteristics of the study group are shown in Table 1.

In addition, patients were divided into subgroups for detailed analysis of response to treatment: (1) patients with a diagnosis of Fetal Alcohol Syndrome (FAS) based on dysmorphia (shortened palpebral fissures, smooth philtrum, thin upper ver-million) and history of alcohol abuse during pregnancy; (2) patients with a genetically and phenotypically confirmed diagnosis of Silver-Russel syndrome (SRS); and (3) patients with other visible dysmorphic features that did not allow a clear diagnosis of FAS or SRS (e.g., triangular skull, hypotelorism, epicanthus, microcephaly, syndactyly, etc.). Patients were also divided into subgroups according to the Tanner scale. The characteristics of each subgroup are presented in Table 1. 

### 2.3. Data Collection

The study included: (a) perinatal data: duration of pregnancy (Hbd), birth weight, SDS birth weight, birth length, SDS birth length, Apgar score, maternal nicotine and/or alcohol abuse during pregnancy, type of hypotrophy and multiple pregnancies; (b) family history: maternal height (Ht), mother’s SDS Ht, father’s Ht, father’s SDS Ht and mean parental height MPH; (c) anthropometric data at baseline: age, sex, Tanner pubertal stage, Ht, SDS Ht, growth rate (HV), weight, body mass index (BMI), bone age (BA), maximum GH concentration [ng/mL] in stimulation tests, insulin-like growth factor-1 (IGF-1) concentration [ng/mL] and rhGH dose [mg/kg/d]; (d) additional history data: features of fetal alcohol syndrome (FAS), confirmed diagnosis of Silver-Russel syndrome (SRS), visible uncharacteristic features of dysmorphia and presence of additional chronic disease. Ht, SDS Ht, HV, body weight, BMI, BA, rhGH dose and IGF-1 levels were reassessed after 12 and 24 months of therapy, respectively. 

Anthropometric data were collected during routine follow-up visits at 12 and 24 months (±3 months) after the beginning of treatment. Height was measured 3 times using a Harpenden stadiometer (with an accuracy of ±0.1 cm), and the arithmetic mean was taken. Bodyweight was measured using certified medical scales (with an accuracy of ±100 g).

### 2.4. Data Analysis

Anthropometric parameters were converted to SDS using the auxological index calculator (by U. Smyczyńska and P. Smyczyńska) and based on Polish centile grids of Warsaw children from 2001 [11]. Ponderal index (PI) = birth weight (g) × 100/birth length [3] (cm^3^) was calculated to assess the type of hypotrophy. PI values < 2.2 were associated with asymmetrical hypotrophy (AH) and PI > 2.2—with symmetrical (SH) [12]. MPH was calculated from the formula MPH = (maternal Ht [cm] + paternal Ht [cm] +13 cm for boys/−13 cm for girls)/2. Bone age was assessed by a qualified radiologist in each clinical center using the Greulich–Pyle method on the basis of a radiogram of the hand and wrist of the non-dominant hand. The stage of puberty was assessed according to the Tanner scale. IGF-1 centile values were calculated from data presented by Bedogni et al. [13].

Unsatisfactory response to treatment was defined by two criteria: (1) the difference in body height (ΔHt) between treatment initiation and follow-up at 12 and 24 months less than 0.3 SDS and (2) ΔHV acceleration of less than 3 cm/year from the pre-treatment growth rate. Patients were divided into groups according to treatment response: poor response (ΔHt SDS < 0.3 and/or ΔHV < 3 cm/year) and good response (ΔHt SDS ≥ 0.3 and/or ΔHV ≥ 3 cm/year). 

### 2.5. Statistical Analysis

Statistical analyses were performed using the R programming and statistical environment (“R environment: a language and environment for statistical computing. R Foundation for Statistical Computing”, Vienna, Austria, version: 4.0.4).

Descriptive statistics were performed to describe individual subgroups by providing: numbers and percentages of individual subgroups for qualitative data and ranges and means for quantitative data. Significance was set at *p* < 0.05.

### 2.6. Ethical and Legal Considerations

The study was conducted in accordance with the Statute of the Department of Pediatrics, Endocrinology, Diabetology, Metabolic Disorders and Cardiology of the Developmental Age (Statute no. WMS-123/01/S/12/2019), approved by Pomeranian Medical University in Szczecin, Poland. The study was observational in nature. Parents of all children treated with rhGH in Poland consented to the treatment prior to its initiation. The study was conducted according to the guidelines of the Declaration of Helsinki. 

## 3. Results

### 3.1. Response to Treatment in the Whole Study Group

All patients improved growth ΔHt SDS by an average of 0.57 during the first 12 months of therapy. In the second year (12–24 months), ΔHt SDS was 0.38, which means that over the 2 years of therapy, the response of the entire study group averaged ΔHt SDS of 0.95.

**Figure 2:**
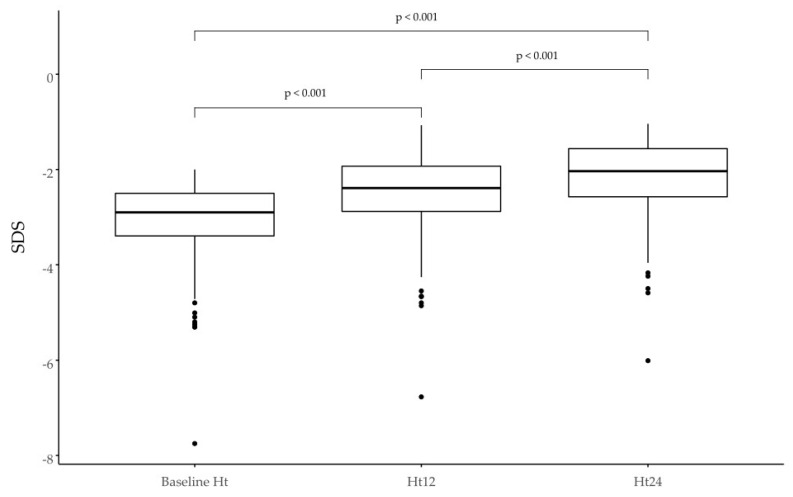
Growth in SGA children is expressed as the change in height (Ht) SD score at the baseline and after 12 and 24 months of treatment.

**Figure 3:**
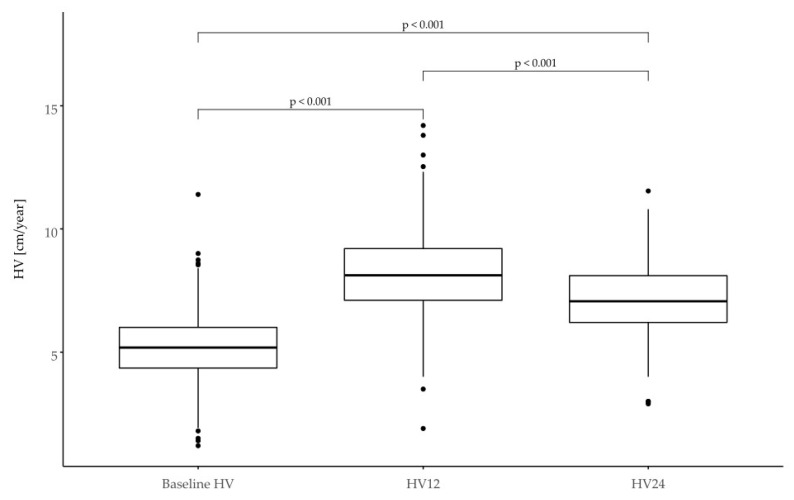
High velocity (HV) in SGA children expressed in cm/year at the baseline and after 12 and 24 months of treatment.

In the group in question, the criterion of poor response to treatment ΔHt SDS < 0.3 after the first year of therapy was met by 39 patients (17%), while in the second year, it was 66 patients (44%). Selected growth parameters are shown in Table 2 and Figure 2 and Figure 3.

The mean difference in growth rate for the entire patient group was ΔHV = 2.94 cm/year in the first year and ΔHV = 1.84 cm/year in the second year. One hundred and twenty-three patients (56%) met the criterion ΔHV < 3 cm/year after the first year of therapy and 99 (73%) in the second year of treatment. The response to treatment in different subgroups is shown in Table 3.

The percentage of patients meeting the criteria for a poor response to treatment in the first year of the study was lower in the subgroup of patients whose puberty was assessed at Tanner stage 1 (1TS) (*n* = 190) ΔHt SDS < 0.3 = 28 (15%) than patients with more advanced puberty—Tanner stage 2 (2TS) (*n* = 32) ΔHt SDS < 0.3 = 7 (22%), with puberty stage 3 (3TS) (*n* = 10) ΔHt SDS < 0.3 = 2 (20%) or with puberty stage 4 (4TS) (*n* = 3) ΔHt SDS < 0.3 = 2 (67%). In the second year, the response to treatment according to pubertal stage was as follows: 1TS (*n*= 132) ΔHt SDS < 0.3 = 54 (41%), 2TS (*n* = 14) ΔHt SDS < 0.3 =10 (71.43%), 3TS (*n* = 5) ΔHt SDS < 0.3 = 2 (50%). After 24 months of therapy in the study group there were no more patients in pubertal stage 4, which means that they completed the treatment at an earlier stage.

Details of the percentages of patients at each pubertal stage meeting the criterion of ΔHV < 3 cm/year can be found in Table 4. A total of two patients (20%) with puberty assessed grade 3 and one patient (33%) assessed grade 4 accelerated growth by at least 3 cm/year, and none of them improved their growth rate beyond the required value during the second year of therapy.

### 3.2. Response to Treatment in Patients with Uncharacteristic Dysmorphic Features

Patients with non-characteristic dysmorphic features constituted the largest subgroup of those listed in the study. 

Patients with dysmorphic features (*n* = 31) improved growth ΔHt SDS by an average of 0.54 SDS during the first year of therapy and by 0.35 SDS during the second year. The mean ΔHV during the first year of therapy was 2.78 cm/year, and during the second year, ΔHV was 1.95 cm/year.

In the study group, the criterion of poor response to treatment ΔHt SDS < 0.3 after the first 12 months of therapy was met by five patients (16%), while in the second year was by seven children (37%). Seventeen patients (59%) met the criterion Δ HV < 3 cm/year after the first year of therapy, and 11 (61%) did not improve their growth rate by at least 3 cm. Response to treatment is shown in Table 3.

### 3.3. Response to Treatment in Patients with FAS

Patients diagnosed with fetal alcohol syndrome had both severe shortage of body length at birth (birth length SDS = −1.77) and body weight (birth weight SDS = −4.24). Patients in this subgroup presented the slowest growth rate before therapy (HV0 = 4.46 cm/year vs. mean HV0 = 5.19 cm/year for the whole study group). 

Patients diagnosed with FAS (*n* = 15) improved growth ΔHt SDS on average by 0.43 SDS during the first year of therapy and by 0.3 6SDS during the second year. The mean ΔHV in the first year of therapy was 3.09 cm/year, and in the second year, ΔHV was 2.28 cm/year.

In the study subgroup, the criterion of poor response to treatment ΔHt SDS < 0.3 after the first year of therapy was met by five patients (33%), while in the second year, it was by three children (43%). 

Seven patients (54%) after the first year of therapy met the criterion of ΔHV < 3 cm/year and 3 (60%) in the second year of therapy. Response to treatment is shown in Table 3.

### 3.4. Response to Treatment in Patients with SRS

Patients diagnosed with Silver-Russel syndrome were smallest at birth (birth weight SDS = −4.43; birth length SDS = −2.28) and had the most pronounced body height deficiency at the start of rhGH treatment (Ht0 SDS = −4.33). Patients in this subgroup were significantly younger at the start of therapy: 84 months (7 years) vs. 108.5 months (9.04 years) for the entire study group. 

Patients diagnosed with SRS (*n* = 9) improved growth ΔHt SDS by an average of 0.74 SDS during the first year of therapy and by 0.72 SDS during the second year. The mean ΔHV during the first year of therapy was 2.59 cm/year, and during the second year, ΔHV was 3 cm/year. 

None of the patients diagnosed with Silver-Russel syndrome met the ΔHt SDS < 0.3 criterion throughout the analysed period. Five patients (62%) during the first 12 months and four patients (67%) during the second year did not accelerate growth by 3 cm/year. Response to treatment is shown in Table 3.

### 3.5. Comparison of Criteria for Evaluating Response to Treatment

In the analysed material, inadequate response to treatment expressed by both adopted criteria was fulfilled by 16% of all patients after the first year and 40% after the second year. Considering any criterion adopted by us, these percentages were significantly higher; 58% of patients after the first and 77% of patients after the second year of treatment, respectively. The values for the different subgroups analysed in the study are shown in Table 5. 

### 3.6. rhGH Dose

The mean initial dose of rhGH was 0.031 mg/kg/d (0.217 mg/kg/week) and remained at a similar level throughout the observation period (Table 1).

### 3.7. IGF-1 Concentration

**Figure 4:**
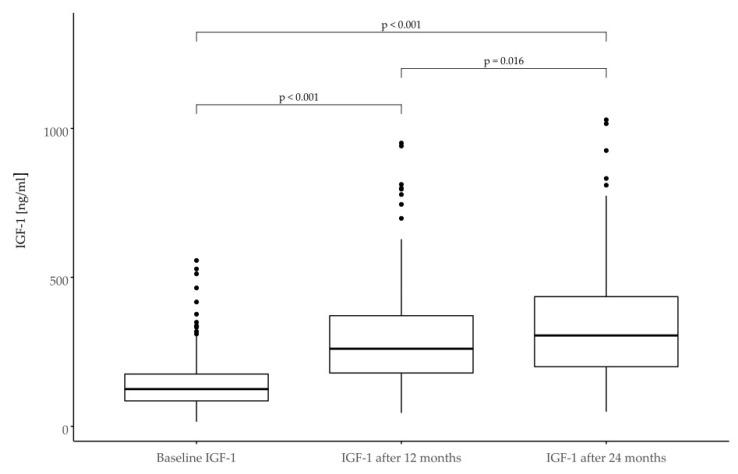
IGF-1 serum concentration values expressed in ng/mL at the baseline and after 12 and 24 months of treatment.

Initially, 14 patients were found to be IGF-1 deficient (value < 3rd percentile for age and sex). During follow-up, most patients were observed to have IGF-1 within the normal range, with few cases where control values were >97th percentile. Detailed data are presented in Table 6 and in Figure 4.

### 3.8. Bone Age

**Figure 5:**
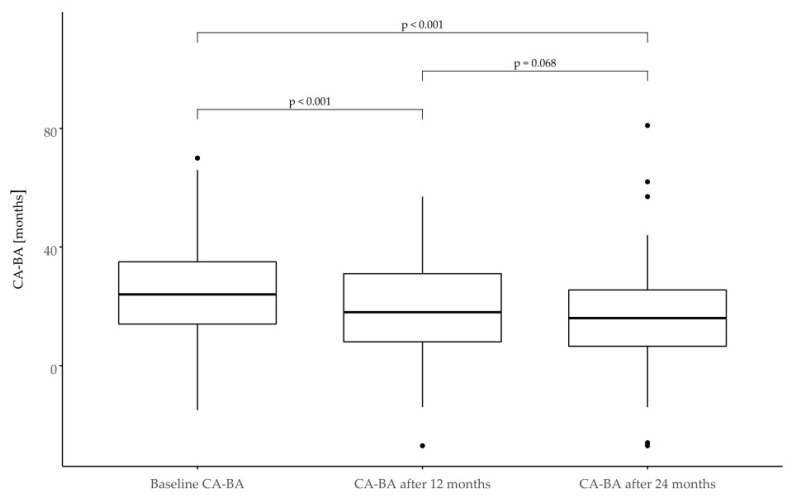
Difference in calendar age and bone age (CA-BA) in SGA children expressed in months at the baseline and after 12 and 24 months of treatment.

**Figure 6:**
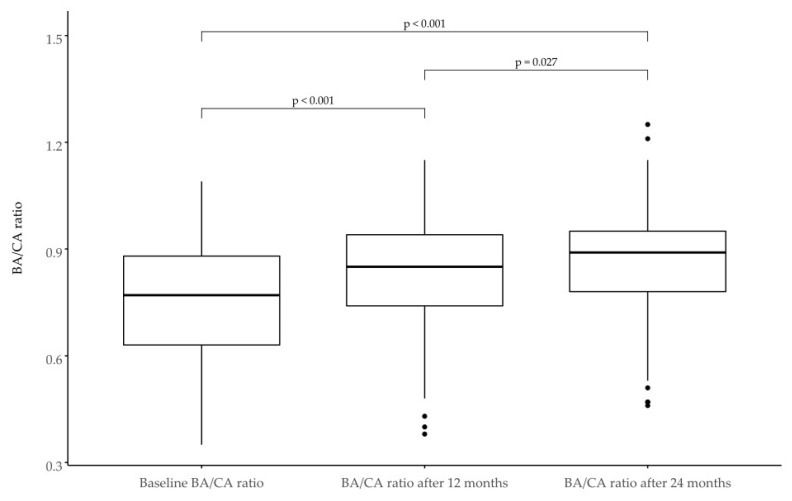
Bone age/calendar age ratio (BA/CA) in SGA children at the baseline and after 12 and 24 months of treatment.

At the baseline, the mean CA-BA was 25 months (2 years 1 month). The BA/CA ratio was initially 0.75 and gradually increased to 0.86. At the end of the follow-up period, a mean CA-BA of 16 months was observed. The biggest initial difference was observed in the subgroup of SRS patients. Detailed information is provided in Table 7 and Figure 5 and Figure 6.

### 3.9. Type of Hypotrophy

In the analysed group, 177 patients presented asymmetrical hypotrophy (76%). Twenty-two patients with uncharacteristic features of dysmorphia were diagnosed with AH (71%), 11 with FAS (79%) and 6 with SRS (67%), respectively.

## 4. Discussion

The primary goal of rhGH therapy for short stature patients with SGA is to improve growth and achieve satisfactory final height. Ongoing molecular and genetic studies demonstrate the complexity of theshort stature and underline the heterogeneity of the patient group of children with SGA [14]. Since the introduction of growth hormone therapy, numerous attempts have been made to establish reliable, reproducible and easy to evaluate by clinicians’ criteria of response to treatment, allowing monitoring and individualisation of therapy. The most commonly adopted criteria are ΔHt SDS or ΔHV [15]. In their work, Bang et al. [7] and Straetemans et al. [5] showed that ΔHV is the best criterion for assessing response to treatment but also has the highest possible measurement error. In our study, ΔHV showed the highest percentage of patients with a poor response, more than twice as high as the accepted criterion of ΔHt < 0.3. In order to reliably assess the growth rate and correctly qualify the patient for rhGH treatment, a detailed growth rate observation is carried out in clinical centres in Poland for at least 6 months prior to the initiation of treatment. Most patients have a full observation period. Some patients, especially adolescents with severe growth deficiency with very low Ht SDS and poor prognosis of final growth, are started on rhGH treatment without a full follow-up period. The height velocity is a strictly age-dependent parameter, so unified cut-off values for all patients are not appropriate in daily practice. It would be advisable to create centile charts assessing HV SDS in the Polish population, analogous to Bakker et al., which would take into account patients diagnosed with SGA, which would greatly improve rhGH therapy [13].

Our results for the whole observed group ΔHt12 SDS 0.57 and ΔHt24 SDS 0.38, as well as HV12 8.15 cm/year after the first and HV24 7.17 cm/year after the second year are consistent with the results obtained by Ranke et al. or López-Siguero et al. [16,17]. Both parameters indicate the benefit of treatment for SGA patients because they improve their centile position as well as maintain a relatively good HV. In particular, López’s 2019 data document almost the same HV as in our study during the first year of treatment and slower during the second. When analysing the percentage of patients meeting the criterion of poor response to treatment defined as ΔHt SDS < 0.3 after the first year of therapy, we obtained a result of 17%, while in the second year, we obtained 44%. When the criterion ΔHV < 3 cm/year was adopted, the values were higher—56% after the first and 73% after the second year, respectively. These values are high and prompt an analysis of the reasons for therapeutic failure. In our opinion, they may result from the high heterogeneity of the SGA patient group. In other work, Ranke et al. found a poor response to treatment ΔHt SDS < 0.3 in 15% of patients [5] and Bang et al. in 25% [7]. The values we obtained are similar, but it must be mentioned that our study group included all patients from the registry, regardless of their pubertal stage, and the studies we cited included only prepubertal patients. An exact comparison of the achieved response to treatment is therefore not possible due to the significant differences in the study groups. 

Our study was supported by the size of the study group (235 children) collected from six university centres for growth hormone treatment. All patients in Poland were qualified for the registry according to a unified scheme and received the same initial dose of rhGH. Dose can be modified according to the IGF-1 levels during the treatment [10]. In addition, all patients in the group analysed by us had scheduled follow-up visits at the same time from the start of treatment (after 12 and 24 months ± 3 months, respectively) and performed the same laboratory tests and anthropometric measurements. 

The age of the child at the start of rhGH therapy is a very well-documented factor influencing the response to treatment. The earlier therapy is started, the better the response and, consequently, higher final growth [18,19]. It is recommended to start rhGH treatment as soon as the child has not shown a spontaneous catch-up process. In the USA, it is advisable to start treatment at the age of 2 years. According to European and Polish guidelines, rhGH treatment of short stature children with SGA should be started after the age of 4 years [18]. In our study, the average age at the beginning of therapy was 9.08 years. In the American Norditropin Studies: Web-Enabled Studies (The ANWSER Program^®^) evaluating 360 children with SGA, the mean age was 8.4 years [19], whereas in a European study based on an analysis of data from the KIGS database, evaluating 1909 patients, the average age of treatment initiation was 9.1 years [20]. The age we obtained is much higher than the recommended one but similar to the average in other European countries. It should also be taken into consideration that until 2015, there was no treatment programme dedicated to patients diagnosed with short stature resulting from SGA in Poland; thus, some children did not have the opportunity to start rhGH treatment at the recommended age. The study group also included those patients who, despite indications for treatment, had to wait for qualification. Based on clinical observations and our own experience, it seems that younger and younger children are being qualified for treatment, which is in line with world guidelines. 

The patients with SRS were enrolled in the treatment programme at the earliest (7.0 years). This demonstrates that patients with a specific diagnosis made in infancy are more vigilantly observed by physicians and more quickly referred to the growth hormone treatment programme. Patients with uncharacteristic dysmorphic features, as well as FAS features, started treatment significantly later than patients with SRS. This may be due to the fact that in patients burdened with another chronic disease or syndrome, different socio-health problems come to the fore, and the body height is a secondary concern for caregivers. Typically, children exposed to alcohol during the fetal period come from families of lower socioeconomic status, and consequently, caregivers are less aware of the treatment options for short stature. 

Patients with dysmorphic features, which did not allow an unambiguous diagnosis of the genetic syndrome described so far, constituted 13% of the entire study group. We believe that this is a group that should be highlighted. It is postulated that every child born SGA should be qualified for possible early intervention and treatment. A diagnostic algorithm in short stature patients with SGA was presented by Finken [14]. Our subgroup included patients with dysmorphic features such as triangular skull, microcephaly, syndactyly, hypotelorism and others that were not associated with FAS or SRS. Based on the results, the authors found no significant difference in response to treatment in this subgroup, but more accurate genetic diagnostics in SGA children should be considered, as more and more conditions with growth impairment are described [21]. Understanding the pathogenesis of short stature in SGA patients with dysmorphia could potentially contribute to optimising treatment for this subgroup or introducing new, dedicated standards for them. 

An attempt was also made to look at the response to treatment based on the type of hypotrophy. Depending on the anthropometric measurements of the newborn and the calculated PI, infants with IUGR can be classified into two groups—with symmetrical hypotrophy (SH) or with asymmetrical hypotrophy (AH) [22]. SH (low birth weight and length, small head circumference) is usually caused by a damaging factor that has been present since the beginning of the pregnancy (e.g., alcohol abuse, smoking, maternal diseases). This group includes patients with FAS. AH is associated with late placental failure or genetic defects (e.g., SRS). In the analysed group, 177 children (76%) presented features of asymmetric hypotrophy. The subgroup of patients with non-characteristic dysmorphic features included 22 patients with AH. According to the available research, the type of hypotrophy does not affect the response to rhGH treatment [23]. Our data seem to confirm previous findings. Nevertheless, understanding the pathogenesis of short stature in SGA patients with dysmorphia could potentially contribute to optimising treatment for this subgroup or introducing new, dedicated standards for them. 

The best absolute response to treatment, both after the first and second year, was observed in the subgroup of patients in pubertal stage 1. However, irrespective of the pubertal stage, the best response was observed in each group during the first year of therapy. This confirms the observation that the introduction of therapy induces a growth spurt in every child, irrespective of the stage of puberty, but with the advancement of puberty, this effect cannot be maintained. Analysis of our data showed that after the second year of treatment, there were no more patients with puberty grade 4, according to Tanner. This indicates that these patients, after the first year of rhGH treatment or during the second year, met one of the exclusion criteria defined in the polish programme (e.g., showed an unsatisfactory treatment effect defined as ΔHV < 2 cm/year or reached a bone age of over 14 years in the case of girls and over 16 years in the case of boys). Seventy-three per cent of patients met the poor response criterion of ΔHV < 3 cm/year after 24 months of therapy.

Depending on the response criterion adopted, the analyses identify different percentages of patients. After the first year of treatment, as many as 58% of patients met at least one criterion for an unsatisfactory response, whereas only 16% of patients met both criteria we adopted. Additionally, it should be emphasised that the “true” non-responders were patients who met both criteria for poor response—16% after the first year and 43% after the second year—these values are distributed similarly in all analysed subgroups, excluding SRS. Although 43% did not meet the good response criterion, it should be noted that they benefit greatly from treatment by improving Ht and HV.

Our observations are similar to the conclusions of the Dutch investigators. We conclude that there is no single optimal criterion to evaluate response to rhGH treatment [5]. After the second year of treatment, 40% of the patients met both criteria for poor response. Table 5 shows the details of the criteria we adopted. 

Based on the analysis of collected data, the mean initial dose of rhGH was 0.031 mg/kg/d (0.217 mg/kg/week) and remained at a similar level throughout the observation period. The acceptable dose of growth hormone for short children with SGA accepted for the Polish population is in the range of 0.023–0.061 mg/kg/d (0.16–0.43 mg/kg/week). A dose of 0.036 mg/kg/d (0.25 mg/kg/week) is recommended as the optimal initial dose [10]. The values of GH that we obtained are within the recommended range.

In their study, which included an analysis of a 6-year follow-up of treatment response in patients treated with two different doses of GH, De Zegher et al., as well as van Pareren et al., showed that a lower dose of GH produces better results in the context of height gain [24,25]. In addition, they conclude that alternate therapy with intermittent higher dose may be effective in very young patients but also in those with advanced puberty and poor response to conventional treatment. Nevertheless, it should be taken into account that higher doses could potentially cause excessive acceleration of bone age.

In our analysis, during the first year, the overall response to treatment remained satisfactory. During the second year, the percentage of patients with an unsatisfactory response increased, which may indicate an insufficient dose of GH in the second year. The data we analysed concerned patients treated between 2016 and 2020—this is information from the first years of the therapeutic programme dedicated to SGA patients in Poland. One of the benefits of this work is the possibility to take a critical look at the current management of therapy and draw conclusions in order to optimise treatment in the following years. Additionally, it is worth noting that initiation of treatment at all centres in Poland with the same dose simplifies the analysis and comparison of the effects.

One of the parameters helping to control the safety of the conducted therapy is the determination of IGF-1 concentration in blood serum and relating it to norms for age and gender. During follow-up, most patients maintained IGF-1 levels within the normal range. In a few cases, an increase in IGF-1 above the normal range determined a reduction in the GH dose.

It was established that the greater the difference between bone age and calendar age (CA-BA), the better the response to treatment [26]. In our study group, the initial difference in bone age was 25 months (a mean of 2 years). It is suggested that children born with SGA advance puberty and bone age faster and therefore present worse growth potential [27]. In our study, the CA-BA was high, and the vast majority of children did not equalise bone age with calendar age. In their analysis, Horikawa et al. [28] found no significant progression of bone age to calendar age during long-term follow-up, which is in line with our findings.

The BA/CA ratio we obtained was comparable to those presented in the work of López-Siguero et al. [17]. During the course of growth BA/CA ratio did not reach the value of 1. This means that patients in the analysed group still had growth potential, and it was reasonable to continue treatment with growth hormone.

Patients with a confirmed diagnosis of Silver-Russel syndrome seem to benefit most from the GH treatment. Suspicion of SRS is most often made in infancy due to profound deficiency in birth weight and/or length [29]. Once the diagnosis of SRS is confirmed, patients are informed that GH treatment may be included after the child is 4 years old, so they remain under long-term observation and are eligible for the registry at the first possible moment. Patients in this subgroup also had the best treatment outcomes. Based on the observations of this group, it is evident that the diagnosis of SGA or IUGR is already at birth; parental education and clinician awareness accelerates the initiation of treatment and therefore maximises the benefits of treatment. Although the children with SRS absolute values of Ht SDS were the lowest, they were the only ones who maintained a continuous improvement of body height, and for them, the final effect of the treatment was most strongly expressed. Despite the small size of the subgroup, we feel it important to point out the outcome of the treatment in those patients. Further studies involving a larger group of patients with SRS are needed to confirm our findings.

### Limitations of Work

All patients were included in the study, irrespective of their pubertal stage or age. However, our analysis serves to critically evaluate the effectiveness of treatment of short stature in children with SGA; hence we included all children treated at six centres in the country.

Another limitation of our study was the incomplete duration of the required 6 month pre-treatment growth observation period in some patients. As in other non-interventional studies, it is not possible to obtain 100% data.

Despite the small size of the subgroup of children with SRS and FAS, it was decided to list their responses as deserving attention. A larger group would have increased the statistical significance of the study.

## 5. Conclusions

Despite careful and detailed qualification of patients for the therapeutic programme, 17% of them do not derive benefit from the first year of therapy and 44% from the second year, taking into account the ΔHt SDS < 0.3 parameter. The criterion ΔHV < 3 cm/year shows a higher percentage of patients with a poor response to therapy (56% in the first year and 73% in the second year, respectively). Despite the high rates of poor response to treatment, both Ht SDS and HV in the entire study group and all of the subgroups were statistically improving in a significant way. Patients with SRS were significantly younger at baseline and showed the best response to treatment, which was sustained throughout the follow-up period. 

Patients before the onset of puberty responded better to treatment compared to the rest of the group. Regardless of the stage of puberty at the start of treatment, the best response was observed after the first 12 months of therapy. 

Patients with fetal alcohol syndrome presented the slowest growth rate before treatment and were eligible for the programme at the latest. 

The study showed that different values are obtained depending on the criterion used to assess response to treatment. The assessment of height velocity generated higher proportions of patients with a poor response. 

Careful monitoring of the therapy provided, an individual approach to the patient, verification of the treatment and a decision on its continuation after one year is essential to optimise treatment effects.

In a majority of the patients, the initial dose of rhGH did not increase levels of IGF-1 > 97th percentile during the 24-month observation. 

In order to maintain a good HV, it is advisable to individualise GH dosage in children with SGA.

## Figures and Tables

**Table 1:** Selected background characteristics of the study group.

Characteristic	All Mean (SD)	Uncharacteristic Features of Dysmorphia Mean (SD)	FAS Mean (SD)	SRS Mean (SD)
*n*	235	31	15	9
Gender				
F	98	13	6	4
M	137	18	9	5
Age at onset of treatment [months]	109 (35)	100 (35)	110 (29)	84 (30)
Tanner stage at onset of treatment				
1	190	27	11	8
2	32	3	4	1
3	10	0	0	0
4	3	1	0	0
Mother’s height, SDS	−1.02 (1.26)	−0.41 (1.84)	−0.53 (0.41)	−0.89 (0.78)
Father’s height, SDS	−0.98 (1.22)	−0.75 (1.17)	−0.68 (1.40)	−0.84 (1.25)
MPH	166 (13)	169 (11)	170.6 (5.1)	168.8 (6.0)
Birth weight, SDS	−3.24 (1.16)	−3.22 (1.12)	−4.24 (1.48)	−4.43 (1.31)
Birth length, SDS	−0.98 (1.54)	−0.96 (1.40)	−1.77 (1.51)	−2.28 (2.34)
GH0 dose [mg/kg/d]	0.031(0.005)	0.032 (0.006)	0.029 (0.005)	0.031 (0.004)
GH12 dose [mg/kg/d]	0.033 (0.005)	0.034 (0.006)	0.032 (0.006)	0.033 (0.004)
GH24 dose [mg/kg/d]	0.030 (0.004)	0.032 (0.004)	0.032 (0.004)	0.034 (0.003)
Ht0, SDS	−3.05 (0.79)	−3.24 (0.86)	−3.00 (0.51)	−4.33 (1.59)
HV before treatment [cm/year]	5.19 (1.45)	5.11 (1.31)	4.46 (1.07)	5.27 (1.85)
BMI0	14.73 (2.05)	14.7 (3.3)	13.95 (1.39)	13.03 (0.85)
BMI12	15.36 (2.39)	15.1 (3.5)	14.67 (1.56)	13.71 (1.62)
BMI24	15.71 (2.55)	15.45 (3.32)	14.31 (1.49)	14.89 (2.24)

Data are expressed as mean (standard deviation). FAS: Fetal Alcohol Syndrome; SRS: Silver-Russel syndrome; SDS: standard deviation score; HV: height velocity; BMI: body mass index.

**Table 2:** Selected growth parameters observed during the treatment.

Characteristic	All Mean (SD)	Uncharacteristic Features of Dysmorphia Mean (SD)	FAS Mean (SD)	SRS Mean (SD)
Ht0 SDS	−3.05 (0.79)	−3.24 (0.86)	−3.00 (0.51)	−4.33 (1.59)
Ht12 SDS	−2.48 (0.78)	−2.70 (0.89)	−2.58 (0.58)	−3.60 (1.56)
Ht24 SDS	−2.19 (0.85)	−2.39 (0.97)	−2.12 (0.56)	−3.32 (1.54)
ΔHt SDS 12-0	0.57 (0.33)	0.54 (0.43)	0.43 (0.31)	0.74 (0.22)
ΔHt SDS 24-12	0.38 (0.33)	0.35 (0.36)	0.36 (0.14)	0.72 (0.21)
ΔHt SDS 24-0	0.96 (0.49)	1.00 (0.54)	0.86 (0.19)	1.48 (0.37)
HV0 [cm/year]	5.19 (1.45)	5.11 (1.31)	4.46 (1.07)	5.27 (1.85)
HV12 [cm/year]	8.15 (1.75)	8.05 (1.87)	7.52 (1.62)	7.92 (1.54)
HV24 [cm/year]	7.17 (1.74)	7.09 (2.17)	7.03 (1.13)	8.20 (1.45)

Data are expressed as mean (standard deviation). FAS: Fetal Alcohol Syndrome; SRS: Silver-Russel syndrome; Ht: height; SDS: standard deviation score; HV: height velocity.

**Table 3:** Response to rhGH treatment in different subgroups.

**After 12 Months**			
**Group**	**N**	**ΔHt SDS < 0.3**	**ΔHt SDS ≥ 0.3**
All	235 (100%)	39 (17%)	196 (83%)
Uncharacteristic features of dysmorphia	31 (100%)	5 (16%)	26 (84%)
FAS	15 (100%)	5 (33%)	10 (67%)
SRS	9 (100%)	0 (0%)	9 (100%)
**After 24 Months**			
**Group**	**N**	**ΔHt SDS < 0.3**	**ΔHt SDS ≥ 0.3**
all	151 (100%)	66 (44%)	85 (56%)
Uncharacteristic features of dysmorphia	19 (100%)	7 (37%)	12 (63%)
FAS	7 (100%)	3 (43%)	4 (57%)
SRS	7 (100%)	0 (0%)	7 (100%)
**After 12 Months**			
**Group**	**N**	**ΔHV < 3 cm/year**	**ΔHV ≥ 3 cm/year**
all	218 (100%)	123 (56%)	95 (44%)
Uncharacteristic features of dysmorphia	29 (100%)	17 (59%)	12 (41%)
FAS	13 (100%)	7 (54%)	6 (46%)
SRS	8 (100%)	5 (62%)	3 (38%)
**After 24 Months**			
**Group**	**N**	**ΔHV < 3 cm/year**	**ΔHV ≥ 3 cm/year**
all	136 (100%)	99 (73%)	37 (27%)
Uncharacteristic features of dysmorphia	18 (100%)	11 (61%)	7 (39%)
FAS	5 (100%)	3 (60%)	2 (40%)
SRS	6 (100%)	4 (67%)	2 (33%)

FAS: Fetal Alcohol Syndrome; SRS: Silver-Russel syndrome; Ht: height; HV: height velocity; SDS: standard deviation score.

**Table 4:** Response to treatment according to pubertal stage.

**After 12 Months**			
**Tanner Stage**	**N**	**ΔHt SDS < 0.3**	**ΔHt SDS ≥ 0.3**
1	190 (100%)	28 (15%)	162 (85%)
2	32 (100%)	7 (22%)	25 (78%)
3	10 (100%)	2 (20%)	8 (80%)
4	3 (100%)	2 (67%)	1 (33%)
**After 24 Months**			
**Tanner Stage**	**N**	**ΔHt SDS < 0.3**	**ΔHt SDS ≥ 0.3**
1	132 (100%)	54 (41%)	78 (59%)
2	14 (100%)	10 (71%)	4 (29%)
3	5 (100%)	2 (40%)	3 (60%)
**After 12 Months**			
**Tanner Stage**	**N**	**ΔHV < 3 cm/** **year**	**ΔHV ≥ 3 cm/year**
1	174 (100%)	103 (59%)	71 (41%)
2	31 (100%)	10 (32%)	21 (68%)
3	10 (100%)	8 (80%)	2 (20%)
4	3 (100%)	2 (67%)	1 (33%)
**After 24 Months**			
**Tanner Stage**	**N**	**ΔHV < 3 cm/year**	**ΔHV ≥ 3 cm/year**
1	117 (100%)	84 (72%)	33 (28%)
2	14 (100%)	10 (71%)	4 (29%)
3	5 (100%)	5 (100%)	0 (0%)

FAS: Fetal Alcohol Syndrome; SRS: Silver-Russel syndrome; Ht: height; HV: height velocity, SDS: standard deviation score.

**Table 5:** Comparison of different growth response criteria to rhGH treatment.

**After 12 Months**			
**Group**	**N**	**ΔHt SDS < 0.3** **or** **ΔHV < 3 cm/year**	**ΔHt SDS < 0.3** **and** **ΔHV < 3 cm/year**
All	218 (100%)	126 (58%)	34 (16%)
Uncharacteristic features of dysmorphia	29 (100%)	18 (62%)	4 (14%)
FAS	13 (100%)	8 (62%)	3 (23%)
SRS	8 (100%)	5 (62%)	0 (0%)
**After 24 Months**			
**Group**	**N**	**ΔHt SDS < 0.3** **or** **ΔHV < 3 cm/year**	**ΔHt SDS < 0.3** **and** **ΔHV < 3 cm/year**
All	136 (100%)	105 (77%)	54 (40%)
Uncharacteristic features of dysmorphia	18 (100%)	11 (61%)	6 (40%)
FAS	5 (100%)	3 (60%)	2 (40%)
SRS	6 (100%)	4 (67%)	0 (0%)

FAS: Fetal Alcohol Syndrome; SRS: Silver-Russel syndrome; Ht: height; HV: height velocity, SDS: standard deviation score.

**Table 6:** Percentile values of IGF-1 in different subgroups.

Characteristic	All Mean (SD)	Uncharacteristic Features of Dysmorphia Mean (SD)	FAS Mean (SD)	SRS Mean (SD)
**IGF-1 Percentile at Baseline**				
<3 percentile	14	4	0	1
3–97 percentile	219	26	14	8
>97 percentile	0	0	0	0
**IGF-1 percentile after 12 months**				
<3 percentile	1	0	0	0
3–97 percentile	222	30	14	9
>97 percentile	8	1	1	0
**IGF-1 percentile after 24 months**				
<3 percentile	1	0	0	0
3–97 percentile	140	17	7	6
>97 percentile	7	1	0	1

Data are expressed as mean (standard deviation). FAS: Fetal Alcohol Syndrome; SRS: Silver-Russel syndrome; IGF-1: insulin-like growth factor 1.

**Table 7:** Bone age, difference between calendar age and bone age, bone age/calendar age ratio in different subgroups.

Characteristic	All Mean (SD)	Uncharacteristic Features of Dysmorphia Mean (SD)	FAS Mean (SD)	SRS Mean (SD)
*n*	235	31	15	9
**BA [months]**				
Baseline	84 (39)	75 (41)	86 (33)	54 (37)
After 12 months	101 (39)	92 (41)	107 (27)	72 (37)
After 24 months	112 (37)	101 (42)	98 (16)	74 (20)
**CA-BA ** **[months]**				
Baseline	25 (15)	25 (17)	24 (14)	30 (14)
After 12 months	19 (15)	20 (18)	15 (16)	24 (12)
After 24 months	16 (16)	18 (18)	14 (16)	20 (17)
**BA/CA ratio**				
Baseline	0.75 (0.16)	0.72 (0.20)	0.77 (0.15)	0.60 (0.20)
After 12 months	0.83 (0.15)	0.80 (0.18)	0.89 (0.13)	0.72 (0.17)
After 24 months	0.86 (0.14)	0.84 (0.19)	0.88 (0.13)	0.78 (0.20)

Data are expressed as mean (standard deviation). FAS: Fetal Alcohol Syndrome; SRS: Silver-Russel syndrome; BA: bone age; CA: calendar age.

## Data Availability

The data presented in this study are available on request from the corresponding author. The data are not publicly available due to ethical restrictions.
